# Effect of TLR2 on the proliferation of inflammation-related colorectal cancer and sporadic colorectal cancer

**DOI:** 10.1186/s12935-020-01184-0

**Published:** 2020-03-30

**Authors:** Shuang Meng, Yingjie Li, Xiaozhen Zang, Zheng Jiang, Huahan Ning, Jing Li

**Affiliations:** 1 Department of Gastroenterology, The First Affiliated Hospital of Jinzhou Medical University, No. 2, Section 5 Renmin Street, Guta District, Jinzhou City, 121001 Liaoning Province China; 2 Jinzhou Medical University, No. 40, Section 3, Songpo Road, Linghe District, Jinzhou City, 121001 Liaoning Province China; 3 Jinzhou No. 2 Hospital, No. 2, Section 6, Nanjing Road, Linghe District, Jinzhou City, 121001 Liaoning Province China

**Keywords:** Inflammation-related colorectal cancer, Sporadic colorectal cancer, Toll-like receptor 2, Cell proliferation

## Abstract

**Background:**

Colitis-associated cancer (CAC) is a complication of inflammatory bowel disease (IBD) with a poor prognosis because it is often diagnosed in advanced stages with local progression or metastasis. Compared with the more common polyp-induced sporadic colorectal cancer (sCRC), CAC has different molecular mechanisms. Toll-like receptor 2 (TLR2) expression is not limited to cells related to inflammation and immune function. High levels of TLR2 expression in tumor tissues of colorectal cancer (CRC) patients have been reported. This report is to investigate the effects of knockout and knockdown of the TLR2 gene on the proliferation of CAC and sCRC.

**Methods:**

Twelve C57BL/6 J wild-type mice (WT) and 12 TLR2 knockout mice (TLR2-/-) were used to rapidly establish a colitis-associated cancer (CAC) model via the 1,2-dimethylhydrazine-dextran sodium sulfate (DMH-DSS) method and were divided into the normal WT control group (NC), TLR2 knockout control group (KC), normal wild-type tumor modeling group (NT), and TLR2 knockout tumor modeling group (KT), with 6 mice in each group. The general performance of the mice during modeling, the gross changes of the colon and the rectum, and the pathological score of HE staining were used to observe tumor growth. The expression of TLR2 was detected by immunohistochemistry, and tumor proliferation was detected by Ki67 labeling. Lentivirus carrying TLR2-RNAi was used to stably infect colorectal cancer cells (HCT116 and HT29) to knock down TLR2 gene expression. The experimental groups included the uninfected control group, negative control group, and gene knockdown group. After infection, the expression of TLR2 protein was detected by Western blot, and cell proliferation and the cell cycle were detected by the CCK-8 method and fluorescence-activated cell sorting. Western blot was used to detect the expression levels of p- NF-κβ, cyclin D1 and cyclin D3 protein in each group of cells.

**Results:**

TLR2 knockout in the CAC model resulted in greater changes in body weight and more severe diarrhea and colorectal hemorrhage. However, knocking out the TLR2 gene reduced the shortening of colorectal length, the number of tumors, and the total tumor volume and inhibited the growth of CAC. Knocking out the TLR2 gene also reduced the pathological score and tumor severity. TLR2 was localized in the cell membrane of the colorectal epithelium of the NC group and of the colorectal tumors of the NT group and was highly expressed in the NT group, while antigen Ki67 was localized in the nucleus of the colorectal tumor cells of the NT group and the KT group, and its expression was reduced in the KT group. In an in vitro sporadic colorectal cancer cell experiment, TLR2 protein in the TLR2 knockdown group was significantly downregulated, and TLR2 knockdown significantly inhibited the proliferation of HCT116 and HT29 colorectal cancer cells, resulting in G1 phase arrest. The expression levels of p-NF-κβ, cyclin D1 and cyclin D3 proteins in TLR2 gene knockdown group cells were significantly reduced.

**Conclusion:**

Knockout and knockdown of TLR2 can inhibit the proliferation of inflammation-related colorectal cancer and sporadic colorectal cancer.

## Background

Colorectal cancer (CRC) is one of the most commonly diagnosed malignant tumors in the world. It is ranked fourth among all male malignancies and third among all female malignancies and is the fourth-leading cause of cancer death in the world. In 2012, the number of new CRC cases was approximately 1.4 million, and the number of deaths was nearly 700,000 [1, 2]. By 2030, the global burden of colorectal cancer is expected to increase by 60%, reaching more than 2.2 million new cases and 1.1 million deaths [2]. Genetic and microenvironmental factors have been identified as key risk factors for CRC, with chronic inflammation being an important risk factor, and up to 20% of cases are associated with chronic infections [3]. Sporadic colorectal cancer (sCRC) is the predominant form of CRC, accounting for more than 90% of CRC cases and occurring in people with no family history of this disease. This type of cancer usually results from continuous accumulation of genetic mutations in genes that control epithelial cell growth and differentiation and is often accompanied by inflammatory manifestations [4, 5]. One type of inflammation occurring before the onset of tumorigenesis is a chronic inflammation caused by immune disorders and autoimmunity. A typical example is inflammatory bowel disease (IBD), which greatly increases the risk of malignancy [6]. Colitis-associated cancer (CAC) is a complication of IBD with a poor prognosis because it is often diagnosed in advanced stages with local progression or metastasis [7]. Compared with the more common polyp-induced sCRC, CAC has different molecular mechanisms [8].

Toll-like receptors (TLRs) are innate immune sensors that recognize a variety of pathogenic components, called pathogen-associated molecular patterns, and can initiate proinflammatory responses to maintain intestinal homeostasis [9]. TLRs not only are an important part of the innate immune system but also activate the adaptive immune system and are widely involved in infectious diseases, inflammatory and allergic diseases, and carcinogenesis [10, 11]. Currently, 10 human TLRs and 13 mouse TLRs have been identified [9]. In the past, most studies have focused on intracellular TLRs such as TLR3, TLR7, and TLR9, but recent studies have shown that cell-surface TLRs, particularly TLR2, also play an important role in the development of autoimmune diseases, and TLR2 expression is not limited to cells related to inflammation and immune function [12]. The expression of functional TLR2 is found in epithelial cells, while TLR2 is also expressed in many tumor cells and tissues [13]. High levels of TLR2 expression in tumor tissues of CRC patients have been reported [10, 14]. It is clear that TLR2 plays an important role in the pathogenesis and development of colorectal cancer. However, the role of TLR2 in CAC and sCRC remains to be further clarified, which may provide promising new targets for CAC and sCRC anticancer therapy.

## Materials and methods

### Animal

TLR2 knockout mice (TLR2−/−) were from the Jackson Laboratory (USA), and C57BL/6 J wild-type mice (WT) were purchased from Beijing HFK Bioscience Co. Ltd. After approval by the Ethics Committee of Jinzhou Medical University, the animals were raised in the specific pathogen-free (SPF)-grade laboratory animal center of Jinzhou Medical University.

### Induction of CAC model

Mice were divided into a normal WT control group (NC), TLR2 knockout control group (KC), normal WT tumor modeling group (NT), and TLR2 knockout tumor modeling group (KT). For the modeling groups, 12 8-week-old female mice (6 TLR2−/− and 6 WT) were intraperitoneally injected with 40 mg/kg of 1,2-dimethylhydrazine (DMH: Sigma, Germany) on days 1, 3, and 5. Starting from the 12th day, the mice received water containing 3% dextran sulfate sodium (DSS: MP Biomedical, USA) for 5 days and then received 12 days of regular drinking water. From the 5th day of DSS water treatment, the mice received injections of 0.9% NaCl at 0.8 ml/day for 3 days. The mice were subjected to 2 DSS cycles, followed by a third cycle in which the mice were given 3% DSS water for 4 days and then regular water for 11 days, and they received injections of 0.9% NaCl on the 4th, 5th, and 6th days of drinking DSS at 0.8 ml/day. For the control groups, 12 8-week-old female mice (6 TLR2-/- and 6 WT) received regular drinking water and injections of an equal amount of 0.9% NaCl at the same time that the modeling groups received the injections of 0.9% NaCl. The general performance of the mice was measured during CAC modeling, including body weight, diarrhea, colorectal bleeding, death, and tumor formation rate.

### Changes in colon in CAC model

After the modeling, the specimens were put under anesthesia. The intestine was cut from the anus to the cecum, and after the feces were removed and put in phosphate-buffered saline (PBS) at 4 °C, the intestine was flattened on the table. The length of the colon and the number of tumors were measured. The long diameter and short diameter of the tumor were measured with Vernier calipers to calculate the tumor volume (tumor volume=1/2 × long diameter × short diameter squared).

### HE staining

The entire colon was washed with PBS and fixed in 4% paraformaldehyde, and paraffin sections were made, followed by hematoxylin and eosin (HE) staining. Pathologists who were blinded to genotypes were asked to score the inflammation and tumor pathological changes. The scoring criteria was as Table 1.

**Table 1:** Pathological scoring criteria of HE staining in mouse colon

Score	Inflammation severity			Tumor pathological	
	Pattern of leukocyte infiltration	Degree of leukocyte infiltration	Number of infiltrated leukocytes	Classification of lesion severity	Histological typing
1 point	Increased lymphoid aggregation	Infiltration confined to the mucosa	Small amount	Intraepithelial neoplasia (nuclear floating does not exceed 3/4 of the total epithelial height)	Highly differentiated adenocarcinoma (> 95% duct formation)
2 point	Cryptitis (neutrophils in the intestinal crypt epithelium)	Infiltration in the submucosa	Medium	Intramucosal carcinoma (disrupted gland structures, with a shared gland wall and a sieve-like structure, without penetrating the mucosal muscle layer)	Moderately differentiated adenocarcinoma (50–95% duct formation)
3 point	Crypt abscess (neutrophils accumulate in the crypt cavity, sometimes showing crypt rupture)/	Infiltration of the whole layer	Severe	Tubular adenocarcinoma (tumor cells penetrating the mucosal muscle layer)	Poorly differentiated adenocarcinoma (0-49% duct formation)
4 point					Undifferentiated carcinoma

### Immunohistochemistry

The paraffin sections were deparaffinized in water, and the antigen retrieval was conducted by heating in a pressure cooker for 10 min. The sections were cooled to room temperature and washed with PBS, followed by the addition of 3% hydrogen peroxide, incubation for 10 min, and washing. The sections were then blocked with blocking solution for 20 min, the blocking solution was discarded, and the sections were added to 1:200 diluted primary antibody (TLR2: abcam, UK, 213676; Ki67: CST, USA, 12202), incubated overnight in a 4 °C refrigerator, and washed with PBS. Then, 1:100-diluted secondary antibodies were added dropwise on the sections and incubated for 30 min at room temperature, followed by a PBS wash. The sections were then added to streptavidin-Peroxidase (streptavidin-POD), incubated at room temperature for 30 min, and washed with PBS. Chromogenic reaction was carried out using 3′-diaminobenzidine (DAB), and the process was observed under the microscope and terminated when the label turned the appropriate color. The DAB staining process usually took 3–5 min, followed by thorough washing in tap water, hematoxylin counterstaining for 2–3 min, full rinsing and differentiation in tap water, dehydration, clarification, and mounting in neutral resin.

### Cell lines

Human CRC cell lines HCT116 and HT29 were purchased from American Type Culture Collection (ATCC; Virginia, USA), passaged within 6 months of thawing, and cultured in McCoy’s 5A medium supplemented with 10% fetal bovine serum and 1% streptomycin (Gibco, USA) in a sterile 37 °C incubator with 5% CO_2_.

### Cell transfection

According to the experimental requirements, the cells in the logarithmic growth phase were counted, plated, and incubated overnight, followed by switching the medium to the infection medium. The optimal amount of lentivirus according to a multiplicity of infection (MOI) = 30 (uninfected control group: no virus; negative control group: RNAi; gene knockdown group: TLR2-RNAi) and 4% transfection reagent (Genechem Group, Shanghai) were added for infection to knock down the TLR2 gene. After incubation for approximately 12–16 h, the medium was then replaced with conventional medium for further culture. The cells were observed to be in good condition, and when the cell infection efficiency was over 80%, the lentivirus-infected cells were further screened by adding 1 μg/mL puromycin (Gibco, USA), and the downstream experiment was carried out after 7–10 more days of culture. Lentivirus carries FITC fluorescent label, and successfully infected cells show green fluorescence under a fluorescence microscope.

### Western blot

After collecting cells in the above uninfected control group, negative control group, and gene knockdown group, the cells were lysed on ice using a lysis buffer containing phenylmethylsulfonyl fluoride (PMSF) for 30 min, then centrifuged at 12,000 rpm for 30 min at 4 °C. The protein content in the supernatant was determined by the bicinchoninic acid (BCA) method to prepare protein samples with the same total protein content. After 10% sodium dodecyl sulfate–polyacrylamide gel electrophoresis, the samples were transferred to polyvinylidene fluoride membranes (Millipore, USA). The membranes were then blocked with 5% fat-free milk for 2 h at room temperature, followed by the addition of the primary antibody (TLR2: CST, USA, 12276S; GAPDH: CST, USA, 5174S; p-NF-κβ: Santa Cruz Biotechnology, USA, 135769; cyclin D1: CST, USA, 55506S; cyclin D3: CST, USA, 2936; β-tubulin: CST, USA, 2128S; β-actin: CST, USA, 3700S)at 1:1000 dilution and incubation at 4 °C overnight. The membranes were then washed with Tris-buffered saline with 0.1% Tween 20 (TBST) 3 times for 5 min per wash, followed by incubation for 2 h at room temperature with secondary antibody at 1:5000 dilution. The membrane was then washed 3 × 5 min with TBST. The protein bands were visualized using the enhanced chemiluminescence (ECL) solution (Azure Biosystems, USA) to detect the expression of protein.

### Cell counting Kit-8 (CCK-8)

The abovementioned HCT116 (2000 cells/well in 96-well plates) and HT29 cells (5000 cells/well in 96-well plates) of the uninfected control group, negative control group, and gene knockdown group were seeded into 96-well plates, and 5 parallel wells were set for each group. Cells were incubated in a 37 °C incubator with 5% CO2. At 0, 24, 48, and 72 h after cell attachment, CCK-8 solution (Dojindo Molecular Technologies, Japan) was added at a ratio of 10 μL/100 μL per well. Cells were placed in a 37 °C incubator with 5% CO_2_ and incubated for 1 h. The absorbance (optical density) values of each group at a wavelength of 450 nm were measured using a microplate reader, and the growth curves were plotted.

### Fluorescence-activated cell sorting (FACS analysis)

The abovementioned uninfected control group, negative control group, and gene knockdown group of cells were seeded in a 6-well plate at 5 × 105/well and incubated for 48 h, followed by cell collection. Cells were washed twice with precooled PBS and resuspended in 300 µl precooled PBS. Subsequently, 700 µl of precooled absolute ethanol was slowly added in drops and mixed well, followed by fixation at 4 °C overnight. The mixture was then centrifuged at 1000 rpm for 5 min, the supernatant was discarded, and the pellet was then washed with precooled PBS 2 times. The cells were resuspended in 500 µl precooled PBS and added to 20 µl of RNase A solution (BestBio, Shanghai), followed by incubation in a 37 °C water bath for 30 min. Cells were then centrifuged again at 1000 rpm for 5 min, the supernatant was discarded, and the cells were resuspended in 500 µl propidium iodide (PI) staining solution (BestBio, Shanghai), followed by staining at 4 °C for 30 min in the dark. FACS analysis was carried out according to standard procedures and analyzed by software of Novo Express 1.1.0.

### Statistical methods

Statistical analysis was performed on experimental data using SPSS 25.0 statistical software. The measurement data are expressed as the mean ± SD. The independent-sample t test was used for comparisons between 2 groups. Analysis of variance (ANOVA) was used for comparisons between 3 or more groups. Fisher’s least significant difference (LSD) test and the Student–Newman–Keuls multiple range (SNK) test were used for pairwise comparison within groups. A difference was considered statistically significant at P < 0.05.

## Results

### General performance

**Fig. 1:**
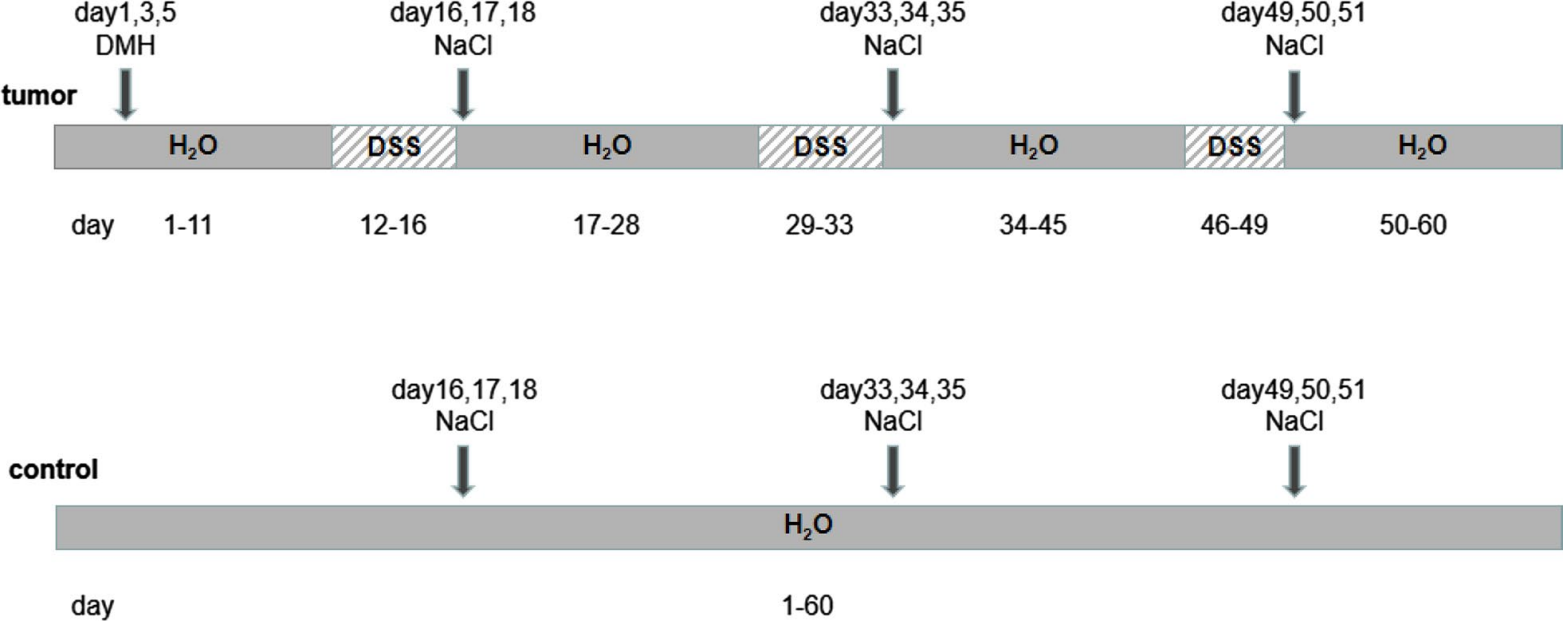
Protocol for the induction of the animal model. Control: WT and TLR2−/− mice without DMH-DSS; tumor: WT and TLR2−/− mice with DMH-DSS

**Fig. 2:**
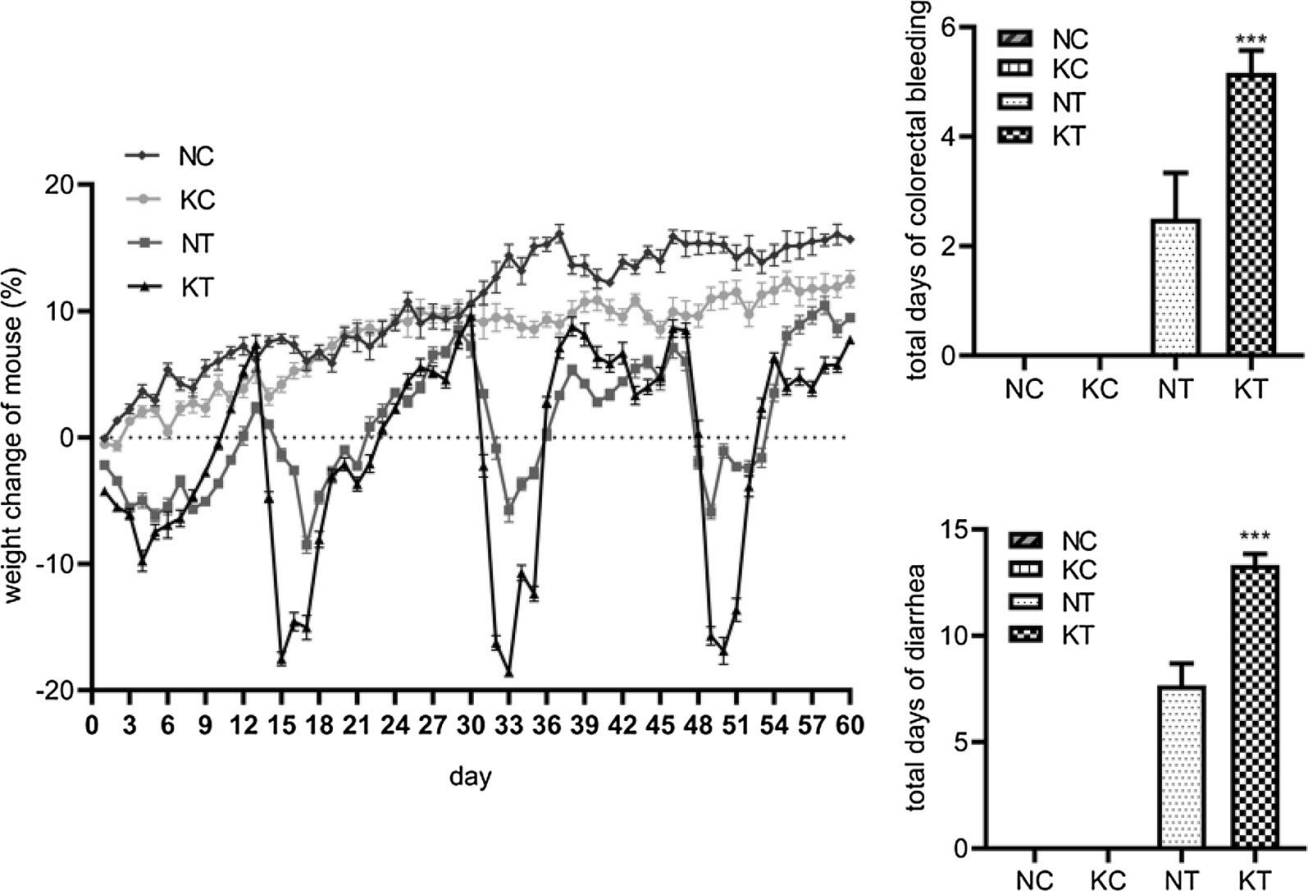
General symptoms such as body weight, total days of diarrhea, and total days of bleeding. NC: WT without DMH-DSS; KC: TLR2−/− without DMH-DSS; NT: WT with DMH-DSS; KT: TLR2-/- with DMH-DSS. ***P < 0.001, KT vs NT group (n = 6)

To verify the role of TLR2 in the CAC tumorigenesis process, we established a working CAC model (Fig. 1). The general performance of the mice is shown in Fig. 2. The body weight showed a trend of increasing during the entire experimental period. The NC, KC, NT and KT groups presented a descending order of weight gain rates. In each cycle of DSS, the body weight of the mice first increased briefly and then decreased. Compared with the NT group, the weight increase and decrease in the KT group were more severe, and the body weights of the NT group and the KT group were significantly lower than those of the NC group and the KC group at the late stage of DSS administration. The rates of mouse diarrhea and rectal hemorrhage were as shown in Fig. 2. In the 3 cycles of DSS administration, the total diarrhea and rectal bleeding durations were significantly increased in the NT group (7.67 ± 1.03; 2.50 ± 0.84) and KT group (13.33 ± 0.52; 5.17 ± 0.41) compared to the NC group (0.00 ± 0.00; 0.00 ± 0.00) and KC group (0.00 ± 0.00; 0.00 ± 0.00), and the duration of diarrhea and duration of colorectal bleeding in the KT group were longer than in the NT group (P < 0.001). The mortality rate of CAC model mice was 0, and the tumor formation rate of CAC model mice was 100%. The above results suggest that DMH+DSS has a good modeling effect, TLR2 knockout can reduce the body weight of mice, and the CAC model also reduces the body weight of mice, while TLR2 gene knockout in the CAC model can lead to more severe changes in the body weight and more severe diarrhea and colorectal bleeding.

### Changes in gross morphology

**Fig. 3:**
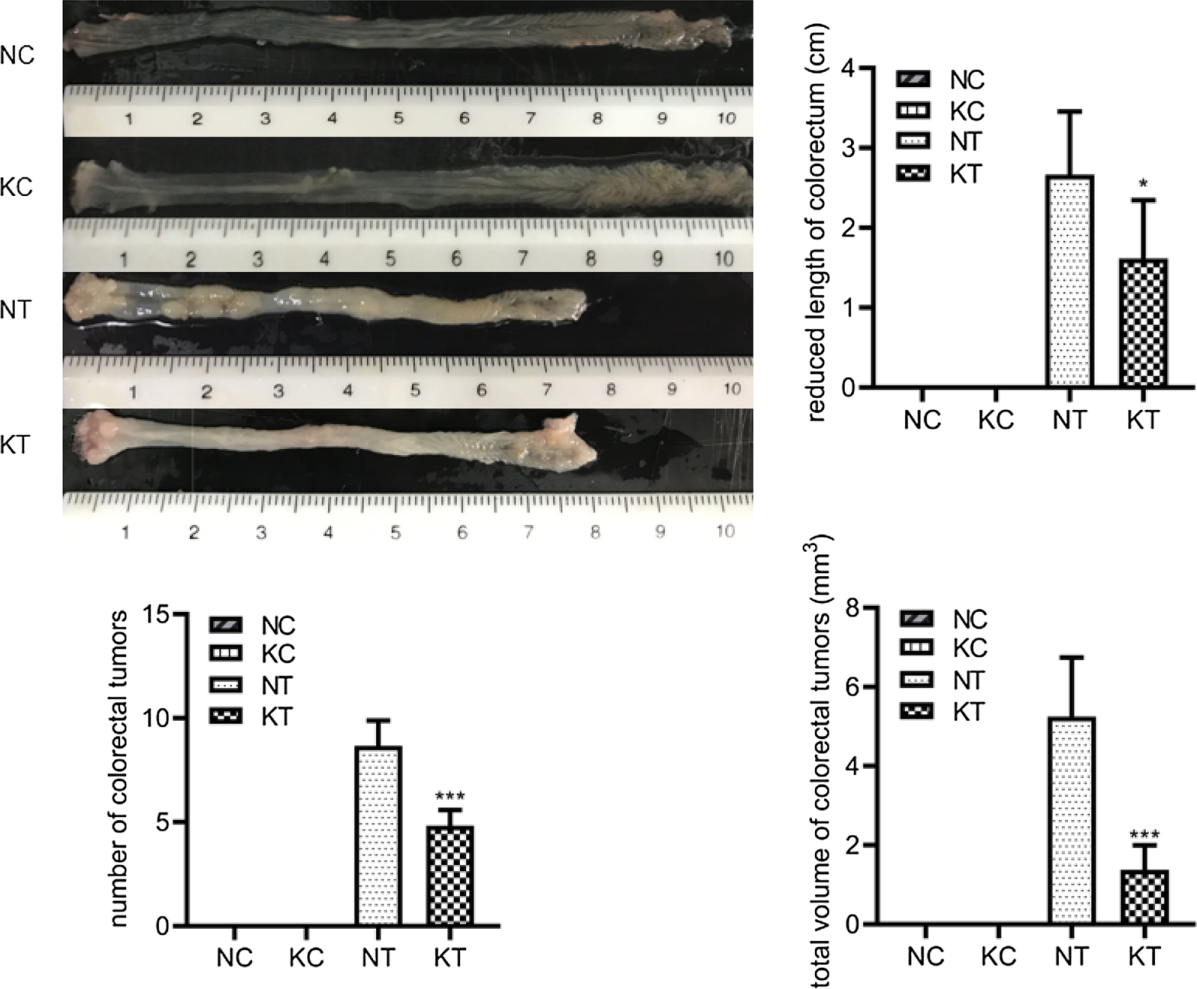
General pathological changes such as shortening of colorectal length, number of tumors, and total volume of the tumor. NC: WT without DMH-DSS; KC: TLR2-/- without DMH-DSS; NT: WT with DMH-DSS; KT: TLR2−/− with DMH-DSS. *P < 0.05, KT vs NT group (n = 6); ***P < 0.001, KT vs NT group (n = 6)

We observed changes in gross morphology after modeling, as shown in Fig. 3. The colorectal length shortening, number of colorectal tumors, and total tumor volume were significantly increased in the NT group (2.67 ± 0.79; 8.67 ± 1.21; 52.48 ± 14.97) and KT group (1.62 ± 0.73; 4.83 ± 0.75; 13.77 ± 6.18) compared to the NC group (0.00 ± 0.00; 0.00 ± 0.00; 0.00 ± 0.00) and KC group (0.00 ± 0.00; 0.00 ± 0.00; 0.00 ± 0.00), while the KT group showed a significant reduction compared to the NT group (P = 0.038; P = 0.00; P = 0.001). These results suggest that knocking out the TLR2 gene can reduce the colorectal length shortening, tumor number, and total tumor volume and inhibit CAC growth.

### HE staining

**Fig. 4:**
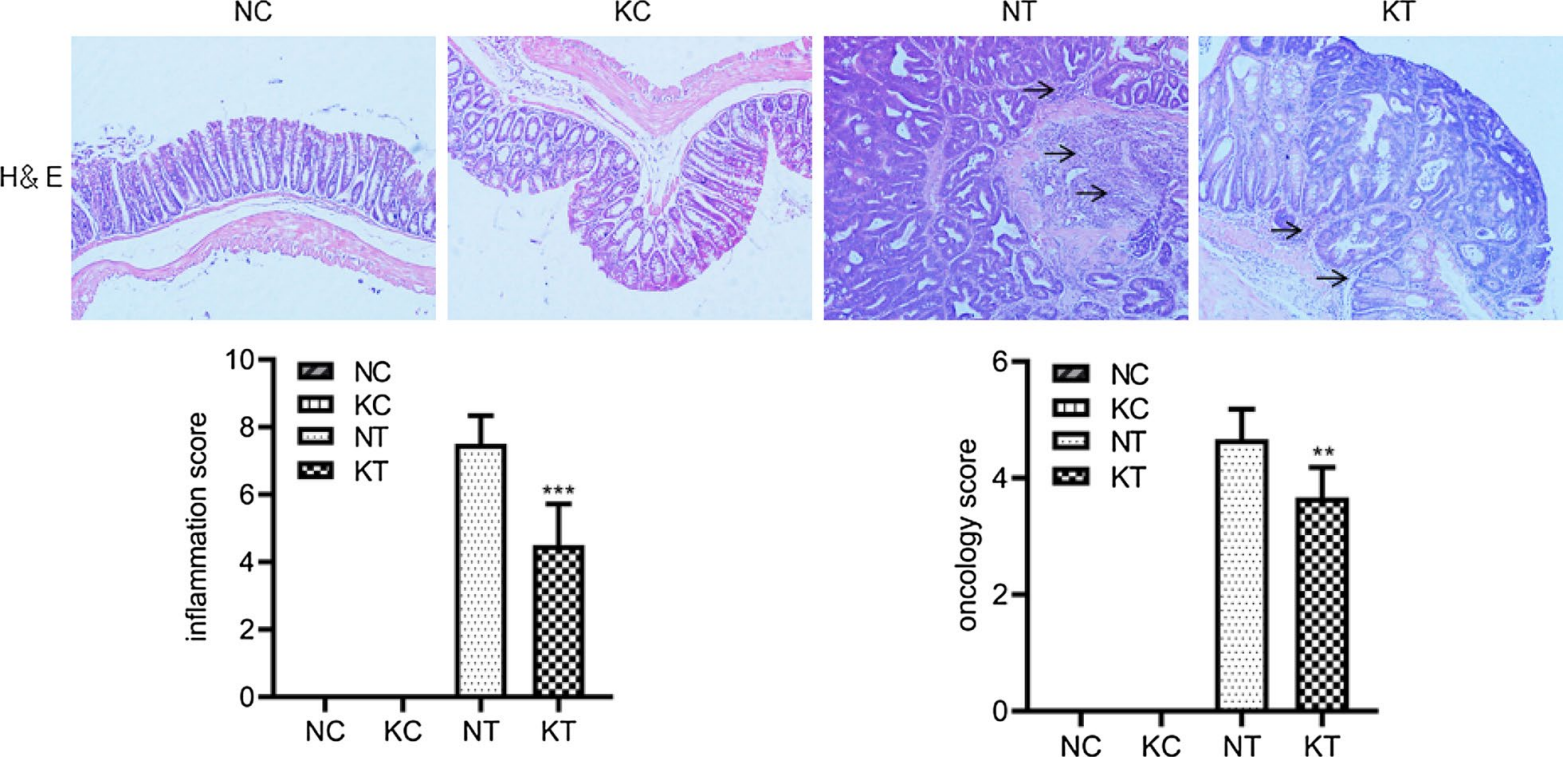
Mouse colorectal HE staining (microscope ×100) and tumor pathological score. NC: WT without DMH-DSS; KC: TLR2−/− without DMH-DSS; NT: WT with DMH-DSS; KT: TLR2−/− with DMH-DSS. Blue represents the nucleus and pink represents the cytoplasm. These arrows represent infiltrating leukocytes. ***P < 0.001, KT vs NT group (n = 6); **P < 0.01, KT vs NT group (n = 6)

As shown in Fig. 4, the pathological scores of HE staining revealed that the inflammation degree score and the tumor pathological score significantly increased in the NT group (7.50 ± 0.84; 4.67 ± 0.52) and the KT group (4.50 ± 1.22; 3.67 ± 0.52) compared with the NC group (0.00 ± 0.00; 0.00 ± 0.00) and the KC group (0.00 ± 0.00; 0.00 ± 0.00), while those in the KT group significantly decreased compared to the NT group (P = 0.001; P = 0.007). The results suggest that knocking out the TLR2 gene can reduce the CAC pathological score and reduce the severity of the tumor.

### Immunohistochemistry

**Fig. 5:**
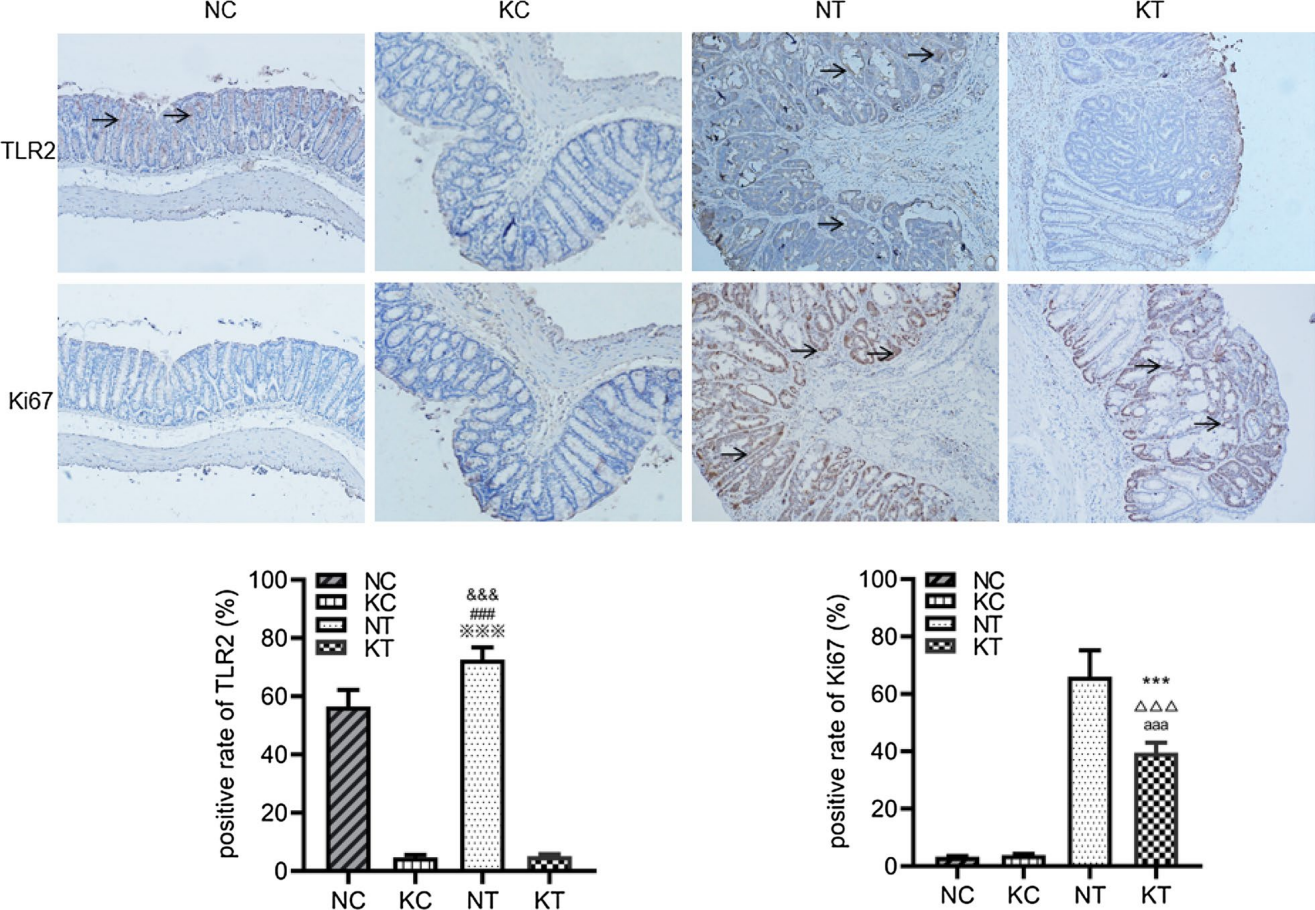
Mouse colorectal immunohistochemistry (microscope ×100) and rate of cells with positive expression. NC: WT without DMH-DSS; KC: TLR2−/− without DMH-DSS; NT: WT with DMH-DSS; KT: TLR2−/− with DMH-DSS. Arrows represent positive staining. ^&&&^P < 0.001, NT vs NC group (n = 6); ^###^P < 0.001, NT vs KC group (n = 6);  ^※※※^P < 0.001, NT vs KT group ( n = 6). ***P < 0.001, KT vs NT group (n = 6); ^△△△^P < 0.001, KT vs NC group (n = 6); ^aaa^P < 0.001, KT vs KC group (n = 6)

The immunohistochemical results (Fig. 5; Table 2) showed that TLR2 was localized on the cell membrane of the colorectal epithelium in the NC group and of the colorectal tumors in the NT group, and the positive rates of the NT group (72.63% ± 4.20%) and NC group (56.57% ± 5.64%) were higher than those of the KC group (4.73% ± 0.82%) and the KT group (5.10% ± 0.65%), while the positive rate of the NT group was higher than that of the NC group (P < 0.001). Ki67 was localized in the nucleus of colorectal tumors in the NT group and the KT group. The Ki67-positive rates of the NT group (66.03% ± 9.21%) and KT group (39.53% ± 3.54%) were significantly higher than those of the NC group (3.30% ± 0.27%) and KC group (3.87% ± 0.41%), and the positive rate of the KT group was significantly reduced compared to that of the NT group (P < 0.001). These findings suggest that TLR2 is highly expressed in CAC and knocking out TLR2 can inhibit CAC proliferation.

**Table 2:** Percentage of positive cells in various groups of colon detected by Immunohistochemistry method (n = 6, ${{\bar{\text{x}}}} \pm {\text{s}}$ )

Group	Percentage of TLR2 positive cells	Percentage of Ki67 positive cells
NC	56.57% ± 5.64%	3.30% ± 0.27%
KC	4.73% ± 0.82%	3.87% ± 0.41%
NT	72.63% ± 4.20%***	66.03% ± 9.21%
KT	5.10% ± 0.65%	39.53% ± 3.54%^∆∆∆^

*** *P* < 0.001 compared NC group; ^∆∆∆^ *P<* 0.001 compared with NT group

### Cell transfection

**Fig. 6:**
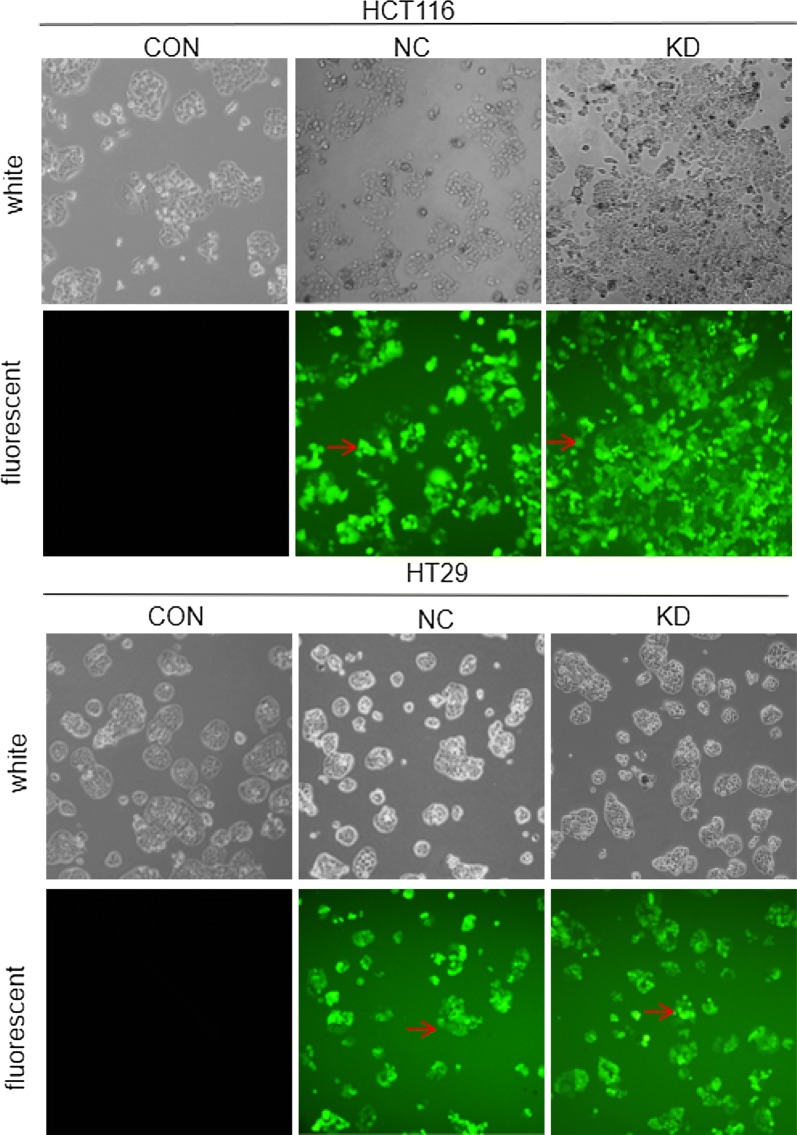
The morphological images of HCT116 and HT29 cells at day 5 after infection with lentivirus under a fluorescence microscope (×40). CON: HCT116 or HT29 cells without lentiviral infection; NC: HCT116 or HT29 cells infected with negative-RNAi lentivirus; KD: HCT116 or HT29 cells infected with TLR2-RNAi lentivirus (RNA interference). Green represents FITC fluorescence

In the process of constructing a strain with stable lentivirus infection, the results of transfection efficiency under the fluorescence microscope (Fig. 6) showed that the transfection efficiency rates of HCT116 and HT29 negative control groups and gene knockdown groups reached more than 80%. There was no difference in transfection efficiency (P > 0.05), and the cells could be used to verify the expression of TLR2 protein.

### Western blot

**Fig. 7:**
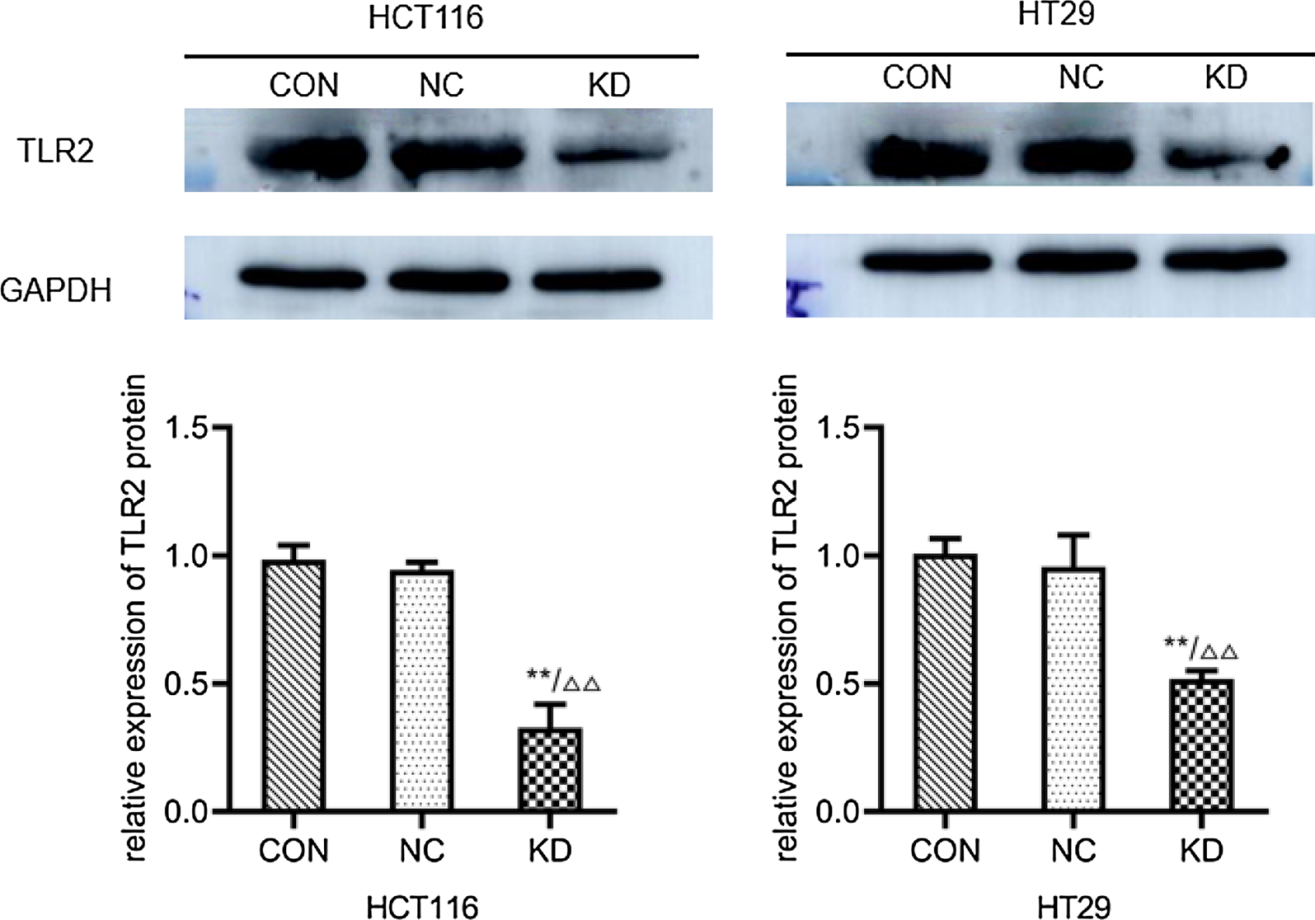
The expression levels of TLR2 protein in HCT116 and HT29 cells infected with lentivirus were detected by Western blotting. CON: HCT116 or HT29 cells without lentiviral infection; NC:  HCT116 or HT29 cells infected with negative-RNAi lentivirus; KD: HCT116 or HT29 cells infected with TLR2-RNAi lentivirus (RNA interference). GAPDH was used as the internal reference. **P < 0.01 KD vs CON group (n = 3); ^△△^P < 0.01 KD vs NC group (n = 3)

**Fig. 8:**
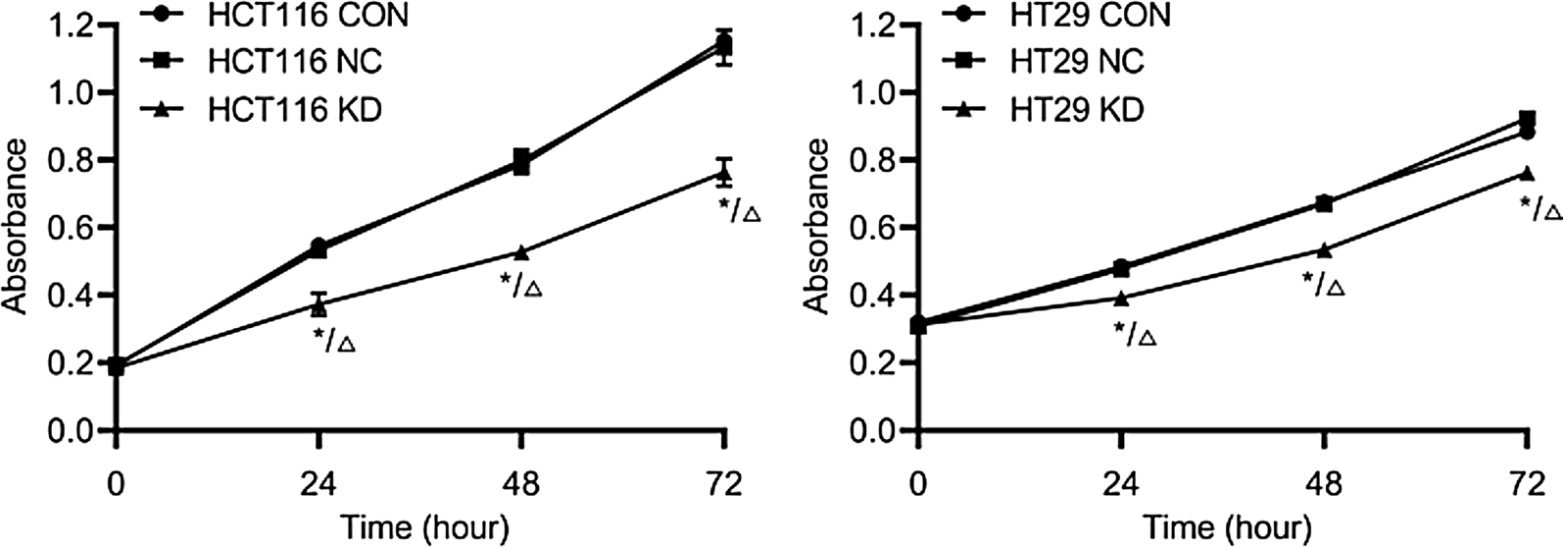
The expression levels of proteins in HCT116 and HT29 cells in various groups detected by Western blotting method. CON: HCT116 or HT29 cells without lentiviral infection; NC: HCT116 or HT29 cells infected with negative-RNAi lentivirus; KD: HCT116 or HT29 cells infected with TLR2-RNAi lentivirus (RNA interference). GAPDH was used as the internal reference. **P < 0.01 KD vs CON group (n = 3); ^△△^P < 0.01 KD vs NC group (n = 3)

The results of Western blotting (Fig. 7) showed that compared with the uninfected control group (0.98 ± 0.06; 1.01 ± 0.06) and the negative control group (0.94 ± 0.03; 0.96 ± 0.12), the expression levels of TLR2 protein in HCT116 and HT29 cells were significantly decreased in the gene knockdown group (0.33 ± 0.09; 0.52 ± 0.03) (P < 0.001). The above results indicated that the lentivirus carrying TLR2-RNAi had a significant downregulation effect on the expression of TLR2 protein in HT29 and HCT116 cells. The results of Western blotting (Fig. 8; Table 3) showed that compared with the uninfected control group and the negative control group, the protein expression levels of p-NF-κβ, cyclin D1 and cyclin D3 in HCT116 and HT29 cells were significantly decreased in the gene knockdown group (P < 0.01).

**Table 3:** Expressions of proteins in HCT116 and HT29 cells in various groups detected by Western blotting method (n = 3, ${{\bar{\text{x}}}} \pm {\text{s}}$)

Group	Expression of protein of HCT116 cells			Expression of protein of HT29 cells		
	p-NF-κβ	cyclin D1	cyclin D3	p-NF-κβ	cyclin D1	cyclin D3
Non-infection control	1.06 ± 0.10	0.89 ± 0.02	1.19 ± 0.12	1.07 ± 0.14	1.03 ± 0.09	1.19 ± 0.05
Negative control	1.02 ± 0.24	0.84 ± 0.07	1.15 ± 0.19	0.90 ± 0.12	0.87 ± 0.10	1.06 ± 0.09
TLR2-RNAi	0.29 ± 0.15**^∆∆^	0.35 ± 0.08**^∆∆^	0.57 ± 0.10**^∆∆^	0.55 ± 0.11**^∆∆^	0.44 ± 0.10**^∆∆^	0.53 ± 0.13**^∆∆^

** *P* < 0.01 compared with non-infection control group; ^∆∆^ *P* < 0.01 compared with Negative control group

### CCK-8

**Fig. 9:**
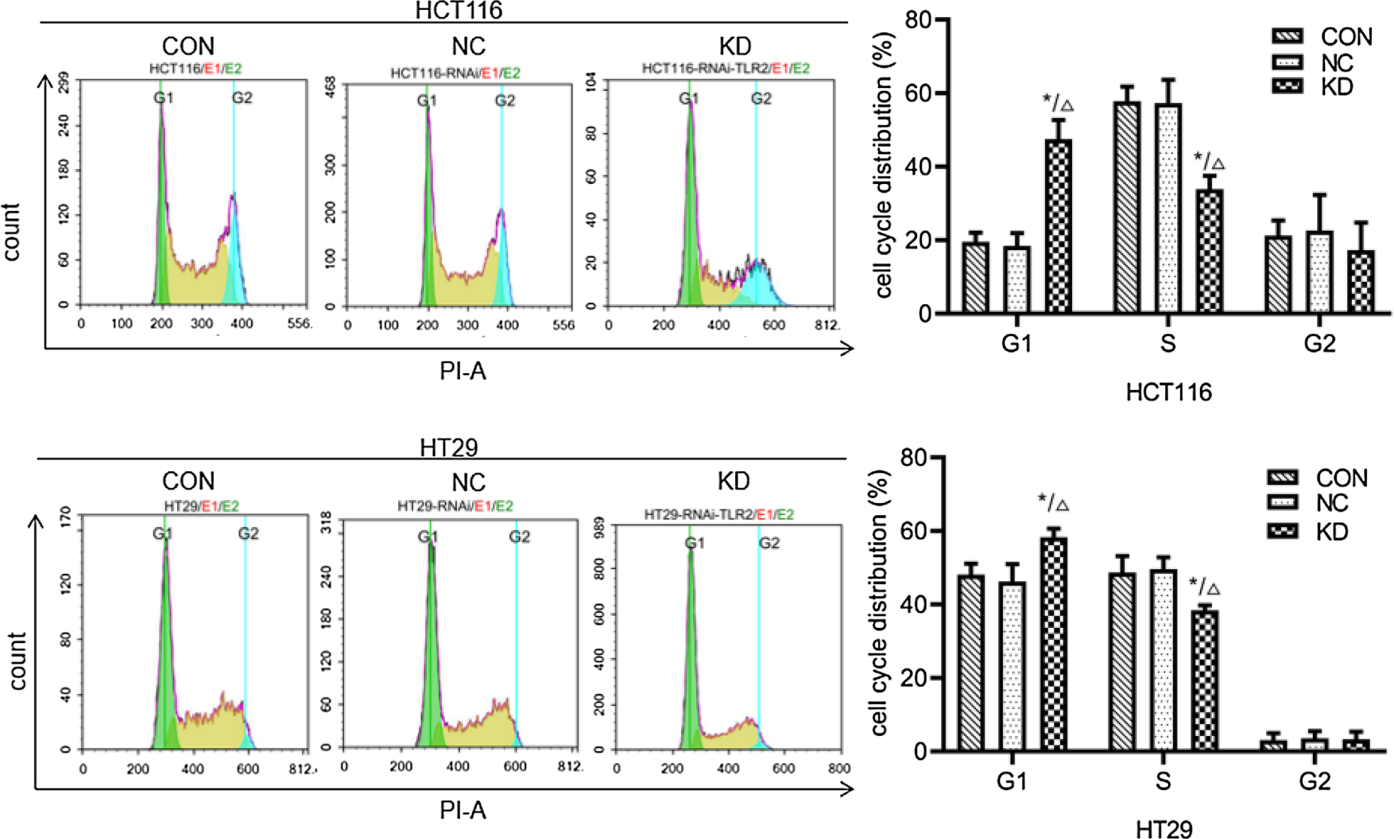
The effect of TLR2 gene knockdown on the proliferation of HCT116 and HT29 cells was determined by CCK-8 assay. CON: HCT116 or HT29 cells without lentiviral infection; NC: HCT116 or HT29 cells infected with negative-RNAi lentivirus; KD: HCT116 or HT29 cells infected with TLR2-RNAi lentivirus (RNA interference). *P < 0.05 KD vs CON group (n = 3); ^△^P < 0.05 KD vs NC group (n = 3)

The results of the CCK-8 assay showed (Fig. 9) that the proliferation rates of HCT116 and HT29 cells in the gene knockdown groups were significantly lower at 24, 48, and 72 h than in the uninfected control group and the negative control group (P < 0.05). These results suggest that knockdown of TLR2 gene expression can significantly inhibit sCRC cell proliferation.

### FACS analysis

**Fig. 10:**
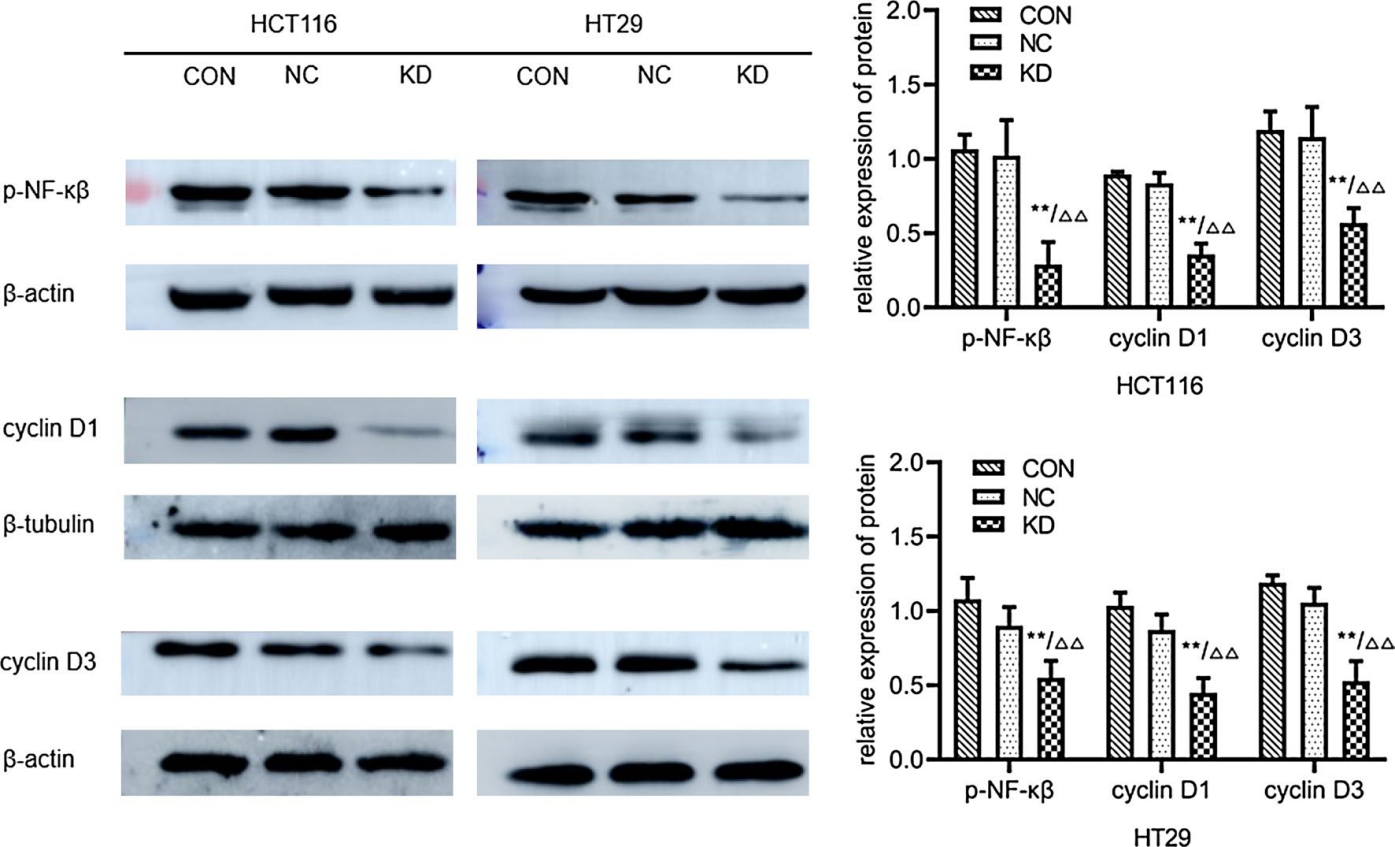
The effect of TLR2 gene knockdown on the cell cycle of HCT116 and HT29 cells was determined by FACS analysis. CON: HCT116 or HT29 cells without lentiviral infection; NC: HCT116 or HT29 cells infected with negative-RNAi lentivirus; KD: HCT116 or HT29 cells infected with TLR2-RNAi lentivirus (RNA interference). *P < 0.05 KD vs CON group (n = 3); ^△^P < 0.05 KD vs NC group (n = 3)

The results of FACS analysis (Fig. 10; Table 4) showed that the percentages of S phase+G2 phase cells in HCT116 and HT29 cells in the knockdown groups were significantly lower than in the uninfected control group and the negative control group (P < 0.05), while the percentages of cells in G1 phase were significantly increased (P < 0.05). This suggests that knockdown of TLR2 gene expression can inhibit the proliferation of colorectal cancer cells, leading to cell cycle arrest in the G1 phase and thereby significantly inhibiting the sCRC cell cycle.

**Table 4:** Cell cycle of HCT116 and HT29 cells in various groups detected by flow cytometry (n = 3, ${{\bar{\text{x}}}} \pm {\text{s}}$)

Group	Cell cycle of HCT116 cells (%)			Cell cycle of HT29 cells (%)		
	G1	S	G2	G1	S	G2
Non-infection control	19.54 ± 2.51	57.86 ± 3.90	21.24 ± 4.12	48.08 ± 3.03	48.67 ± 4.49	3.07 ± 1.91
Negative control	18.40 ± 3.51	57.27 ± 6.37	22.65 ± 9.68	46.21 ± 4.74	49.64 ± 3.16	3.58 ± 1.94
TLR2-RNAi	47.52 ± 5.18*^∆^	33.92 ± 3.60*^∆^	17.33 ± 7.44	58.28 ± 2.31*^∆^	38.40 ± 1.35*^∆^	3.28 ± 2.11

* *P* < 0.05 compared with non-infection control group; ^∆^ *P* < 0.05 compared with Negative control group

## Discussion

TLR2 is a key regulator of the innate immune response and has been shown to play an important role in cancer. In previous studies, TLR2 was found to be expressed at a high level in most patients with gastric cancer, and high expression of TLR2 was associated with proliferative genes and indicated a poor prognosis, while other studies have also suggested that TLR2 promotes the development of gastric cancer [15, 16]. In pancreatic cancer and breast cancer, TLR2 promotes the proliferation of tumor cells [17, 18]. In mantle cell lymphoma, TLR2 promotes the proliferation of tumor cells, and it can also inhibit the cell cycle progression of mantle cell lymphoma, leading to G1 phase arrest [19]. Colorectal cancer tissues usually have higher TLR2 gene expression levels than normal colorectal mucosa from the same patient, and activation of TLR2 and TLR4 in previous studies of colorectal cancer has also been shown to promote tumor cell proliferation [9, 10, 14, 20]. Our study demonstrates that knocking out the TLR2 gene inhibits CAC growth, reduces tumor severity, and reduces tumor proliferation and that TLR2 is highly expressed in CAC, consistent with the reported effects of TLR2 on tumors. In the cellular experiment, we used the CCK-8 assay to find that knockdown of TLR2 can inhibit the proliferation of sCRC cells, and we also found that it inhibited the cell cycle progression of sCRC cells, leading to G1 phase arrest.

In CRC, CAC has a molecular mechanism that is not identical to that of sCRC. The time and frequency of gene mutations in CAC appear to be different from those in sCRC. The frequency of aneuploidy in CAC is higher than in sCRC, and the total methylation level of CAC is lower than in sCRC. Different molecular changes in CAC and sCRC play different roles in tumor repair, immune response, cellular metabolism, and interactions with microbiota during tumorigenesis. These molecules (immunoglobulin light chain V-region locus1 (Igl-V1), galectin 2 (LGALS2), Nur77 dependent gene-1 (NDG1),et al.) play different roles in DNA damage repair, immune and inflammatory responses, cell cycle, apoptosis, and cell metabolism [8, 21, 22]. This study demonstrates that TLR2 plays a common role in tumor proliferation in CAC and sCRC.

Many different drug regimens induce the CAC model, which have different rates of tumor formation and different times of tumor onset. Azoxymethane (AOM) + DSS, DMH + DSS, AOM/DMH + trinitrobenzenesulfonic acid are the 3 most commonly used modeling methods [23–25]. Although DSS + AOM is widely used, AOM is expensive and highly toxic and has high transportation requirements. We therefore have chosen DMH + DSS, which has the same modeling mechanism as DSS + AOM. Based on our previous studies, we adjusted the DMH dose and gave rehydration treatment, thus obtaining the modeling results of 100% tumor formation rate and 0 fatality rate. In our study, the results of animal experiments in terms of body weight and clinical symptoms were not completely consistent with the pathological results. It is possible that TLR2 has a greater impact on body weight, that it interacts with the modeling drug, and that changes in body weight and related symptoms do not directly reflect the severity of the cancer [26].

## Conclusion

Thus, we demonstrated that Knockout and knockdown of TLR2 can inhibit the proliferation of CAC and sCRC through animal experiments and cellular experiments, which suggests that TLR2 plays a key role in CRC and may provide promising new targets for CRC therapy.

## Data Availability

The datasets generated and analysed during the current study are available from the corresponding author on reasonable request.

## Abbreviations

CAC: Colitis-associated cancer
IBD: Inflammatory bowel disease
CRC: Colorectal cancer
sCRC: Sporadic colorectal cancer
TLRs: Toll-like receptors
DMH: 1,2-Dimethylhydrazine
DSS: Dextran sodium sulfate
AOM: Azoxymethane
PMSF: Phenylmethylsulfonyl fluoride
BCA: Bicinchoninic acid
ECL: Chemiluminescence
PI: Propidium iodide
Igl-V1: Immunoglobulin light chain V-region locus1
LGALS2: Galectin 2
NDG1: Nur77 dependent gene-1
